# Passive smoking and risk of oesophageal and gastric adenocarcinomas

**DOI:** 10.1038/sj.bjc.6605023

**Published:** 2009-04-07

**Authors:** L Duan, A H Wu, J Sullivan-Halley, L Bernstein

**Affiliations:** 1 Division of Cancer Etiology, Department of Population Sciences, City of Hope National Medical Center, 1500 East Duarte Road, Duarte, CA 91010, USA; 2 Department of Preventive Medicine, Keck School of Medicine, University of Southern California, 1441 Eastlake Avenue, Los Angeles, CA 90089-9175, USA

**Keywords:** passive smoking, oesophageal adenocarcinoma, gastric adenocarcinoma, case-control study, cigarette smoking, polychotomous logistic regression

## Abstract

Few studies have examined the association between passive smoking and the risk of oesophageal and gastric adenocarcinomas. In a population-based case–control study with 2474 participants in Los Angeles County, there was no evidence that passive smoking had any appreciable effect on oesophageal or gastric adenocarcinomas.

Tobacco smoking is a well-established cause of oesophageal squamous cell carcinoma (ESCC) and oesophageal adenocarcinoma (EA) (IARC, 2004). Generally, the risk for both types of oesophageal cancer increases with increasing duration of smoking and remains high for a number of years after smoking cessation (IARC, 2004). The literature on smoking and stomach cancer also shows a consistent association with cigarette smoking in both men and women (IARC, 2004; Ladeiras-Lopes *et al*, 2008). Alcohol drinking is thought to be associated with ESCC (Freedman *et al*, 2007), but not with EA (Wu *et al*, 2001), and is not directly related to gastric cancer (Chow, 1999). Although much attention has been focused on the association between active smoking and cancer risk, less has been paid to the association between passive smoking and cancer risk, with the exception of lung cancer (IARC, 2004). A few studies have investigated gastric cancer in relation to passive smoking (Hirayama, 1984; Sandler *et al*, 1989; Nishino *et al*, 2001; Mao *et al*, 2002), but none has reported on EA.

We investigated the relationship between passive smoking and the oesophageal and gastric adenocarcinoma (EGA) risks in a population-based case–control study in Los Angeles County.

## Materials and methods

The details of the study population and design have been described elsewhere (Wu *et al*, 2001). Briefly, 1716 eligible patients with newly diagnosed first-incident EA, gastric cardia adenocarcinoma (GCA), or distal gastric adenocarcinoma (DGA) between 1992 and 1997 were identified and contacted for participation through the population-based, Los Angeles County Cancer Surveillance Program. Neighbourhood controls were matched individually to each case patient on sex, race, and age (±5 years). To increase statistical power, we sought two controls for each case whenever possible. In-person interviews were conducted using a structured questionnaire to gather information on demographics, smoking status, smoking history, and household passive smoking exposure during childhood and adulthood. Intensity of passive smoking exposure was measured by the number of smokers who smoked in the participant's presence for at least 1 year and the duration of each exposure. These smokers included the participant's spouse, parents, siblings, or other relatives. Next-of-kin (NOK) were interviewed when case patients were unable to be interviewed due to death or illness. However, we were unable to interview 769 patients who were too ill or died and had no NOK available for interview, whose physicians denied permission to contact, or who refused to participate or could not be located. After excluding participants due to missing information, a total of 938 (220 EA/277 GCA/441 DGA) case patients and 1356 control participants are included in the analyses presented. Age, sex, and race distributions did not differ between the case patients we interviewed and those we did not. Next-of-kin accounted for 269 of the 938 interviews with case patients (65 EA/85 GCA/119 DGA).

We obtained odds ratios (ORs) and 95% confidence intervals (CIs) using polychotomous logistic regression, which allowed us to evaluate ORs for the different cancer sites simultaneously. We adjusted for age, gender (female *vs* male), body mass index (BMI) (kg m^−2^), and ethnicity (others *vs* non-Hispanic white). For validity purposes, we repeated all statistical analyses excluding NOK data. Risk estimates were not materially different from the results based on all subjects combined (i.e., self-respondents and NOK respondents).

## Results

The mean ages of case patients at diagnosis were 62.2 years (s.d.=9.2) for EA patients, 58.7 years (s.d.=12.9) for GCA patients, and 58.0 years (s.d.=12.5) for DGA patients; control participants were, on average, 57.0 years old (s.d.=12.5) on their reference dates (date identified). Among control participants, 60.3% were male, compared with 81.4% of EA patients, 63.4% of GCA patients, and 37.4% of DGA patients. Non-Latino whites represented 72.1% of EA patients, 63.4% of GCA patients, and 24.1% of DGA patients. Oesophageal adenocarcinoma and GCA patients tended to have greater BMI than control participants. Considering all 2294 participants, 769 (33.5%) never smoked any cigarette or other tobacco (i.e., never smoked at least one cigarette a day for 6 months or longer), 1050 (45.8%) were ex-smokers, and 475 (20.7%) were current smokers.

Current smokers were at increased EA risk (adjusted OR, 3.27; 95% CI: 1.56–6.86), increased GCA risk (adjusted OR, 2.07; 95% CI: 1.16–3.69), and increased DGA risk (adjusted OR, 1.83; 95% CI: 1.13–2.99) relative to never smokers who had no passive smoke exposure (Table 1). Never smokers exposed to passive smoking during childhood were not at increased risk of adenocarcinomas of the oesophagus, gastric cardia, or distal stomach compared with never smokers with no passive smoke exposure. The risks did not differ between participants who were exposed to passive cigarette smoke only and those who were also passively exposed to other tobacco products (e.g., cigar or pipe). Exposure to at least one smoker during adulthood was associated with an elevated risk for EA (adjusted OR, 1.80; 95% CI: 0.81–4.00); similar results were observed for duration of exposure. Those exposed to passive smoking as adults whether for fewer, or for more, than 12 person-years were at increased risk for EA and DGA, although the CIs were wide and included 1.0. Trend test indicated a dose–response effect for DGA (*P*_trend_=0.03). No other associations were observed.

## Discussion

We found no evidence that exposure of persons who have never actively smoked to passive smoke during their childhood years or during their adult years strongly influences their risk of EGA. We found nonsignificantly elevated risks of EA and DGA for adult passive smoke exposure.

Direct cigarette smoking plays an important role in the development of oesophageal and gastric cancers (IARC, 2004), for which laboratory studies provide some potential mechanisms. In animal models, cigarette smoke exposure significantly decreased serum epidermal growth factor (EGF) levels (Ma *et al*, 1999). Although the mechanisms by which cigarette smoke exposure decreases serum EGF are still unknown, depletion of EGF has been associated with reduced gastric blood flow, which in turn, results in the promotion of apoptosis in the gastric mucosa (Ma *et al*, 1999; Wang *et al*, 2000). The contents of cigarette smoke may form DNA adducts and induce mutations in tumour suppressor genes (Shin and Cho, 2005). Tobacco smoking may also increase the risk of EA by reducing lower oesophageal sphincter pressure, thereby promoting reflux disease (Dua *et al*, 1998; Pandolfino and Kahrilas, 2000). Using this study population, we have shown earlier that current cigarette smokers have increased risk for EA, GCA, and DGA, and that the deleterious effect on the oesophagus remained for at least 20 years after smoking cessation (Wu *et al*, 2001). In these earlier analyses, the reference group combined never smokers with passive smoke exposure and those without such exposure.

Few studies have examined the association between passive smoking and the risk of gastric adenocarcinoma (Hirayama, 1984; Sandler *et al*, 1989; Jee *et al*, 1999; Nishino *et al*, 2001; Mao *et al*, 2002). In a Japanese cohort study, after 16 years of follow-up, elevated risks of lung cancer were observed in non-smoking women whose husbands smoked (Hirayama, 1984). Although similar risk increases were also observed for nasal sinus cancer, brain tumours, and cancer overall, none of these associations were statistically significant; no associations were noted specifically for oesophageal or gastric cancer (Hirayama, 1984). Whether passive smoking at home affected cancer incidence among non-smoking Japanese women was investigated by linking cohort and cancer registry data (Nishino *et al*, 2001). The results, after 9 years of follow-up, indicated that a husband's smoking might increase the non-smoking woman's risk of ‘smoking-related cancer’ overall (i.e., oral, oropharyngeal, hypopharyngeal, oesophageal, pancreatic, laryngeal, lung, bladder, or renal pelvis cancer), but not significantly. Further, a husband's smoking status was unrelated to the non-smoking woman's risk of gastric cancer. No results were presented for oesophageal cancer. A limitation of this study is that household members’ smoking status was collected only at baseline. In a Canadian population-based case–control study of stomach cancer that separated cancer subsite (cardia *vs* distal), passive smoke exposure was positively associated with gastric cardia cancer risk in a dose–response manner among male never smokers (Mao *et al*, 2002). In contrast, we observed a potentially increased risk of DGA, but not GCA, associated with passive smoke exposure among non-smokers; we were unable to perform the stratified analyses by gender because of the small number of never smokers.

The findings of our study, the first to evaluate passive smoke exposure and risk of EGA in a western population, are essentially negative, but are limited by the small number of never smokers. Larger studies and more precise exposure estimates are needed for more definitive conclusions.

## Figures and Tables

**Table 1:** Adjusted ORs and 95% CIs for active and passive smoking history by cancer site, Los Angeles county

		**Adenocarcinoma of oesophagus**		**Adenocarcinoma of gastric cardia**		**Adenocarcinoma of distal gastric**	
**Exposure to smoking**	**Control**	***n***	**OR^a^ (95% CI)**	***N***	**OR^a^ (95% CI)**	***n***	**OR^a^ (95% CI)**
*Lifetime exposure to smoke (active and passive)*							
No active, no passive	122	12	—	17	—	38	—
No active, passive exposure in childhood only	151	6	0.48 (0.17–1.42)	20	0.89 (0.44–1.79)	34	0.85 (0.48–1.53)
No active, any passive exposure in adulthood^b^	207	22	1.49 (0.65–3.40)	32	0.86 (0.44–1.67)	85	1.30 (0.79–2.14)
Ex-smoker	639	107	1.55 (0.76–3.19)	127	1.07 (0.61–1.86)	177	1.28 (0.82–2.01)
Current smoker	236	70	3.27 (1.56–6.86)	79	2.07 (1.16–3.70)	90	1.82 (1.12–2.97)
							
*Passive exposure type among never smokers*							
No passive smoke exposure	122	12	—	17	—	38	—
Exposure to cigarette only	298	23	1.05 (0.46–2.39)	41	0.79 (0.42–1.48)	97	1.04 (0.64–1.70)
Exposure to other types of tobacco^c^	61	4	0.67 (0.19–2.35)	11	0.87 (0.36–2.10)	22	1.25 (0.61–2.55)
							
*No. of persons who smoke in households of never smokers*							
Childhood							
No childhood exposure	178	20	—	24	—	70	—
1	206	13	0.58 (0.27–1.26)	23	0.76 (0.40–1.42)	51	0.72 (0.45–1.14)
2+	93	5	0.47 (0.15–1.52)	20	1.36 (0.67–2.77)	23	0.82 (0.45–1.49)
Adulthood							
No adult exposure	273	18	—	37	—	72	—
1	141	14	1.80 (0.81–4.00)	20	0.72 (0.37–1.42)	58	1.38 (0.86–2.21)
2+	66	6	1.34 (0.46–3.93)	12	1.04 (0.49–2.22)	27	1.23 (0.66–2.29)
							
*Duration of passive smoke exposure among never smokers*							
Adulthood							
No adult exposure	273	18	—	37	—	72	—
<12 person-exposure years	112	10	1.54 (0.64–3.72)	11	0.55 (0.25–1.20)	34	1.15 (0.67–1.97)
⩾12 person-exposure years	95	10	1.77 (0.73–4.32)	21	1.08 (0.54–2.16)	51	1.54 (0.92–2.58)
*P* for trend^d^			0.43		0.60		0.03

CI=confidence interval; OR=odds ratio.

a ORs from polychotomous logistic regression models, adjusted for age, gender, body mass index at reference age, and ethnicity (others *vs* non-Hispanic white).

b Might or might not be exposed during childhood.

c Other types of tobacco include cigar, pipe, or a combination of these.

d Trend statistics was calculated using continuous measure of exposed duration in person-exposure years.

## References

[bib1] Chow W (1999) Human cancer. Int J Cancer 81: 871–876 10362132 10.1002/(sici)1097-0215(19990611)81:6<871::aid-ijc6>3.0.co;2-#

[bib2] Dua K, Bardan E, Ren J, Sui Z, Shaker R (1998) Effect of chronic and acute cigarette smoking on the pharyngo-upper oesophageal sphincter contractile reflex and reflexive pharyngeal swallow. Gut 43: 537–541 9824582 10.1136/gut.43.4.537 PMC1727281

[bib3] Freedman ND, Abnet CC, Leitzmann MF, Mouw T, Subar AF, Hollenbeck AR, Schatzkin A (2007) A prospective study of tobacco, alcohol, and the risk of esophageal and gastric cancer subtypes. Am J Epidemiol 165: 1424–1433 17420181 10.1093/aje/kwm051

[bib4] Hirayama T (1984) Cancer mortality in nonsmoking women with smoking husbands based on a large-scale cohort study in Japan. Prev Med 13: 680–690 6536942 10.1016/s0091-7435(84)80017-1

[bib5] IARC (2004) IARC Monographs on the evaluation of the carcinogenic risk of chemicals to human. In Tobacco Smoke and Involuntary Smoking. Volumes 83. Lyon, France PMC4781536 15285078

[bib6] Jee SH, Ohrr H, Kim IS (1999) Effects of husbands’ smoking on the incidence of lung cancer in Korean women. Int J Epidemiol 28: 824–828 10597977 10.1093/ije/28.5.824

[bib7] Ladeiras-Lopes R, Pereira AK, Nogueira A, Pinheiro-Torres T, Pinto I, Santos-Pereira R, Lunet N (2008) Smoking and gastric cancer: systematic review and meta-analysis of cohort studies. Cancer Causes Control 19: 689–701 18293090 10.1007/s10552-008-9132-y

[bib8] Ma L, Wang HY, Chow JY, Cho CH (1999) Cigarette smoke increases apoptosis in the gastric mucosa: role of epidermal growth factor. Digestion 60: 461–468 10473971 10.1159/000007692

[bib9] Mao Y, Hu J, Semenciw R, White K (2002) Active and passive smoking and the risk of stomach cancer, by subsite, in Canada. Eur J Cancer Prev 11: 27–38 11917206 10.1097/00008469-200202000-00005

[bib10] Nishino Y, Tsubono Y, Tsuji I, Komatsu S, Kanemura S, Nakatsuka H, Fukao A, Satoh H, Hisamichi S (2001) Passive smoking at home and cancer risk: a population-based prospective study in Japanese nonsmoking women. Cancer Causes Control 12: 797–802 11714107 10.1023/a:1012273806199

[bib11] Pandolfino JE, Kahrilas PJ (2000) Smoking and gastro-oesophageal reflux disease. Eur J Gastroenterol Hepatol 12: 837–842 10958210 10.1097/00042737-200012080-00002

[bib12] Sandler DP, Comstock GW, Helsing KJ, Shore DL (1989) Deaths from all causes in non-smokers who lived with smokers. Am J Public Health 79: 163–167 2913834 10.2105/ajph.79.2.163 PMC1349926

[bib13] Shin VY, Cho CH (2005) Nicotine and gastric cancer. Alcohol 35: 259–264 16054988 10.1016/j.alcohol.2005.04.007

[bib14] Wang H, Ma L, Li Y, Cho CH (2000) Exposure to cigarette smoke increases apoptosis in the rat gastric mucosa through a reactive oxygen species-mediated and p53-independent pathway. Free Radic Biol Med 28: 1125–1131 10832074 10.1016/s0891-5849(00)00207-0

[bib15] Wu AH, Wan P, Bernstein L (2001) A multiethnic population-based study of smoking, alcohol and body size and risk of adenocarcinomas of the stomach and esophagus (United States). Cancer Causes Control 12: 721–732 11562112 10.1023/a:1011290704728

